# Development and evaluation of a questionnaire to measure the perceived implementation of the mission statement of a competency based curriculum

**DOI:** 10.1186/1472-6920-12-109

**Published:** 2012-11-07

**Authors:** Thomas Rotthoff, Martin Stefan Ostapczuk, Judith de Bruin, Klaus-Dietrich Kröncke, Ulrich Decking, Matthias Schneider, Stefanie Ritz-Timme

**Affiliations:** 1 Deanery of study, Medical Faculty, Heinrich-Heine-University, Duesseldorf, Germany; 2 Department for Endocrinology and Diabetes, University Hospital, Düsseldorf, Germany; 3 Institute of Experimental Psychology, Heinrich-Heine-University, Duesseldorf, Germany; 4 Institute of Biochemistry and Molecular Biology I Medical Department, Heinrich-Heine-University, Duesseldorf, Germany; 5 Polyclinic for Rheumatology, University Hospital, Duesseldorf, Germany; 6 Institute for Forensic Medicine, University Hospital Duesseldorf, Duesseldorf, Germany

## Abstract

**Background:**

A mission statement (MS) sets out the long-term goals of an institution and is supposed to be suited for studying learning environments. Yet, hardly any study has tested this issue so far. The aim of the present study was the development and psychometric evaluation of an MS-Questionnaire (MSQ) focusing on explicit competencies. We investigated to what extent the MSQ captures the construct of learning environment and how well a faculty is following - in its perception - a competency orientation in a competency-based curriculum.

**Methods:**

A questionnaire was derived from the MS “teaching” (Medical Faculty, Heinrich-Heine University Düsseldorf) which was based on (inter-) nationally accepted goals and recommendations for a competency based medical education. The MSQ was administered together with the Dundee Ready Education Environment Measure (DREEM) to 1119 students and 258 teachers. Cronbach’s alpha was used to analyze the internal consistency of the items. Explorative factor analyses were performed to analyze homogeneity of the items within subscales and factorial validity of the MSQ. Item discrimination was assessed by means of part-whole corrected discrimination indices, and convergent validity was analyzed with respect to DREEM. Demographic variations of the respondents were used to analyze the inter-group variations in their responses.

**Results:**

Students and teachers perceived the MS implementation as “moderate” and on average, students differed significantly in their perception of the MS. They thought implementation of the MS was less successful than faculty did. Women had a more positive perception of educational climate than their male colleagues and clinical students perceived the implementation of the MS on all dimensions significantly worse than preclinical students. The psychometric properties of the MSQ were very satisfactory: Item discrimination was high. Similarly to DREEM, the MSQ was highly reliable among students (α = 0.92) and teachers (α = 0.93). In both groups, the MSQ correlated highly positively with DREEM (*r* = 0.79 and 0.80, *p* < 0.001 each). Factor analyses did not reproduce the three areas of the MS perfectly. The subscales, however, could be identified as such both among teachers and students.

**Conclusions:**

The perceived implementation of faculty-specific goals can be measured in an institution to some considerable extent by means of a questionnaire developed on the basis of the institution’s MS. Our MSQ provides a reliable instrument to measure the learning climate with a strong focus on competencies which are increasingly considered crucial in medical education. The questionnaire thus offers additional information beyond the DREEM. Our site-specific results imply that our own faculty is not yet fully living up to its competency-based MS. In general, the MSQ might prove useful for faculty development to the increasing number of faculties seeking to measure their perceived competency orientation in a competency-based curriculum.

## Background

A mission statement (MS) sets out long-term goals of an institution in terms of strategies, culture and philosophy [1,2]. Specifically, it should specify the framework for an orientation common to all members of the organization and guide them in making decisions for the benefit of the organization, in motivating themselves and others to put corporate goals into practice, and in furthering identification with the organization (*corporate identity*) as well as constructive communication [3]. The link between a successfully implemented MS and enhanced organizational performance is well known in for-profit organizations [4]. Despite some misgivings about applying such a specifically for-profit tool to a non-profit situation [5], more and more universities have developed their own MSs [1]. Nearly all universities in the United States have formulated MSs, and 80% of them revise their MSs regularly [6].

In addition to MSs applying to the university as a whole, some institutions have specific MSs for their medical faculty. These are often broken down into specific MSs for “teaching”, “research”, “patient care”, etc. [7]. In our faculty, there are also different mission statements. This study pertains to the MS “teaching” of the medical faculty. MSs usually comprise ideals [2]. Once formulated, the question arises to what extent MSs are actually put into practice [8]. One way to approach this question could be an analysis of discrepancies between nominal and actual conditions, which may point out potential areas in which a faculty can work to further the goals defined in the MS.

As a corollary, an MS can be seen as a means of measuring the educational environment of a faculty [9]. In particular, in a “teaching” MS, it may be supposed that the educational climate in the faculty varies positively with the degree to which faculty members take note of the MS and put it into practice; but to date hardly any study has tested this issue. In turn, ascertainment of the educational climate is regarded as a necessary first step towards implementing a reformed curriculum [10,11].

The goal of the present work was the development and psychometric evaluation of a method in the form of a questionnaire derived from an MS focusing on explicit competencies which can enable a faculty to assess how well it is following its own MS - in its perception - and to what extent it makes a statement about the learning environment.

## Methods

Currently, our faculty is working on a radical reform for a competency based medical curriculum. The present curriculum is still organized in a traditional way with a 2-year preclinical course (basic sciences), followed by a 4-year clinical course. The first state examination follows the preclinical course and the second state examination concludes the clinical course. The MS “teaching” of the medical faculty was developed by a working group consisting of 8 academic teachers, 3 medical students and the 3 deans of study. The development was based on (inter-)nationally accepted goals and recommendations for a competency based medical education and practice (national regulation licences for doctors in Germany 2002, Dutch Blueprint 2 (Netherlands, 2009), CanMeds (Canada, 2005), The Scottish Doctor 3 (Scotland, 2009), Tomorrows Doctors (UK, 2009) Swiss Catalogue of learning objectives (Switzerland, 2008) and Catalogue of learning objectives of the Brown University (USA, 2009). Subsequently, a Delphi process was accomplished in the faculty. All faculty members, including the student representatives could participate in the process. Change requests were discussed in the official, elected body of the faculty and accepted or rejected by vote. The MS was officially passed by the faculty in 2009 (Table 1).

**Table 1:** Mission Statement ”Teaching“ of the Medical Faculty, Heinrich-Heine-University, Düsseldorf, Germany

**The Medical Faculty of the Heinrich-Heine University, Düsseldorf is a community of learners and teachers, which develops in livelily interaction and mutual esteem. The teachers actively support the personal and professional development of the students, whose personal initiative is encouraged and demanded. The learners support the teachers in developing their areas of expertise.**	
**Our graduates**	• know the physical, mental and social dimensions of health and disease,
	• master basic medical competencies,
	• make differential diagnoses and develop treatment strategies independently,
	• master basics of scientific work,
	• think critically in consideration of evidence as well as (in the clinic) in consideration of the patients individuality and make professional decisions on that basis,
	• act in consideration of ethical principles,
	• communicate appropriately, sensitively and respectful with patients and colleagues,
	• know their personal limits and cope straight and adequately with errors,
	• have competencies in self-organization and time management,
	• consider health-economic conditions,
	• impart their knowledge to others and,
	• are well prepared for lifelong learning and to develop personally,
**Our teachers**	• are persons in charge, in a position of trust and role models for the students,
	• are competent both didactically as well as in terms of content and are willing to develop perpetually,
	• are in a livelily dialogue with the students and other teachers,
	• provide stimulating feedback and
	• receive recognition for their work by students and faculty.
**Our curriculum**	• encourages the students on a professional and personal level,
	• is patient-oriented, problem-based and interdisciplinary,
	• promotes scientific thinking and working,
	• consists of a core curriculum an offers comprehensive electives,
	• provides scope for academic qualification and for stays abroad,
	• inspires the students for a self-directed learning,
	• is family-friendly and considers the equality of women and men,
	• is accompanied by educational research and
	• is designed and developed jointly by teachers and students.

### MS questionnaire and DREEM

To assess the perceived degree to which current practice reflects the objectives stipulated by the MS, each requirement specified in the MS was transformed into a questionnaire item. The MS comprises the three areas “graduates” (12 objectives), “teachers” (5 objectives) and “curriculum” (9 objectives). Based on these 26 requirements, 37 items were formulated according to general rules of item construction such as only one statement per item, short sentences, quantifiable and specific statements, no double negation, etc. [12,13]. The fact that there are more items (37) than requirements (26) is a result of these rules: some requirements (e.g. “*The teachers are persons in charge and in a position of trust for the students, and role models for the students*”) had to be separated into more than one item (“*The teachers are persons in charge and in position of trust for the students*” and “*The teachers are role models for the students*”).

In addition, the *Dundee Ready Education Environment Measure* (DREEM) [14] was used to assess how well the construct “educational climate” is measured by the questionnaire derived from the MS. Following the usual procedure in constructing and validating a questionnaire, it was assumed that a high positive correlation between the DREEM and the MS questionnaire indicates that it does, indeed, measure educational climate [15]. To be consistent with DREEM items and to enhance clarity, the word “curriculum” was replaced by “course”. To emphasize the ongoing process, the term “graduates” in the MS was changed to “students”.

The DREEM which we applied together with our questionnaire is a culturally non-specific tool measuring the perception of educational environments by students in the health professions. The current version consists of 50 items [16]. Responses to each item are on a scale from 0 (“*strongly disagree*”) to 4 (“*strongly agree*”), the maximum score is thus 200 points. The items encompass five subscales: perception of teaching, perception of teachers, academic self-perception, perception of atmosphere, and social self-perception. Up to now, only two studies have administered DREEM to both students and teachers, in order to detect potential perceptional discrepancies between these groups [17,18].

The questionnaire used in the present study comprised 87 items, all of which were scaled in agreement with the DREEM scale from 0 to 4, yielding a total maximum of 348 points (200 from DREEM + 148 for the MS questionnaire). To preclude potential distortions, respondents did not know which item belonged to which scale [19,20]. Item order was randomized in each questionnaire.

### Participants

The survey was conducted online at the end of the summer semester 2010; 2034 students and 1294 faculty members were contacted by e-mail. Participation was voluntary, data were entered and stored anonymously.

In sum, questionnaires from 1119 students (55.0% return rate; average age 24.1 years, SD = 3.8; 66.0% women) and 258 faculty members (average age 41.7 years, SD = 9.5; 27.9% women) were available for analysis. As it was not clear how many faculty members are actually involved in teaching, the questionnaires were sent to all of the 1294 scientific personnel. The accompanying letter was addressed simply to “teachers” and there were demographic questions concerning teaching activity. It may therefore be legitimately assumed that questionnaires were returned by those who are or had been active in teaching. Due to the initial uncertainty, however, no statement concerning the actual return rate in the faculty sample can be made.

### Data analysis

To assess the perceived degree of implementation of the MS, we defined five categories for the MS questionnaire:

0-29 points: poor implementation.

30-59 points: slight implementation.

60-89: points: moderate implementation.

90-119 points: good implementation.

120-148 points: excellent implementation.

All data were analyzed using the Statistical Package for the Social Sciences (SPSS) 17.0 for Windows. Effect sizes were computed with G*Power [21]. Between-group differences (for example students vs. faculty) were assessed by independent-samples t-tests. Differences in the means of more than two groups (for example teachers in the preclinical course vs. teachers in the clinical course vs. teachers in both courses) were analyzed using analysis of variance. Whenever significant differences were found, pairwise group comparisons were conducted using Tukey’s posthoc-test. Pearson’s correlations were computed to assess associations. For the analysis of nominal-scale data (for example the frequency distribution of the different interpretation categories) χ^2^-tests were used. The interpretation of effect sizes follows Cohen’s [22] criteria: for t-tests, the effect size *d* ≥ 0.80 implies a large effect, *d* ≥ 0.50 a moderate and *d* ≥ 0.20 a small effect. For η^2^ derived from analysis of variance: η^2^ ≥ 0.14 implies large effect, η^2^ ≥ 0.06 moderate and η^2^ ≥ 0.01 small effect. The product-moment correlation coefficient *r* is itself a measure of effect size, with *r* ≥ 0.50 large effect, *r* ≥ 0.30 moderate and *r* ≥ 0.10 small effect. In χ^2^-tests, the measure of effect size is *w*, with *w* ≥ 0.50 large effect, *w* ≥ 0.30 moderate and *w* ≥ 0.10 small effect.

### Psychometric analyses

Item and test analysis involved studying item means and discrimination indices, as well as reliability and validity of the test. Items with a mean < 2 were considered to indicate an area requiring improvement in DREEM [23,24]. The part-whole-corrected discrimination index was considered to be very good for *r* > 0.50 and acceptable for *r* > 0.30 [25].

Evaluation of test reliability was limited to the analysis of internal consistency. Concerning validity, particular emphasis was placed on convergent validation with respect to the German version of DREEM [18] and to demographic criteria (for example phase of studies, previous training). To examine the factorial validity of the MS questionnaire, an explorative factor analysis was conducted. We used the Kaiser-Meyer-Olkin (KMO) value (preferably > 0.60) and Bartlett’s test of sphericity (preferably significant) to assess the suitability of data for factorisation [26]. Apart from theoretical expectations, the Kaiser-Guttman criterion [26] and the screen test [27] were employed as criteria for the extraction of factors.

## Results

### Implementation of the MS

**Figure 1:**
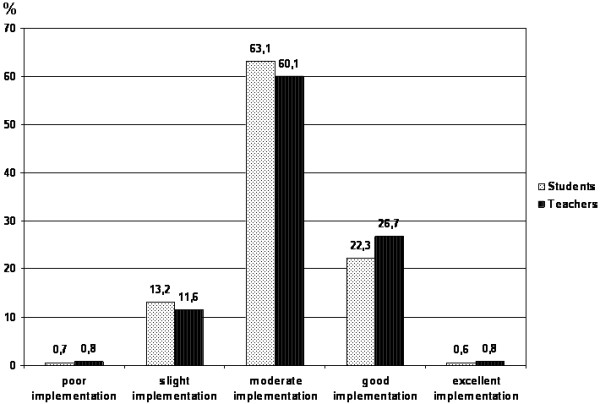
Distribution of responses to the Mission-statement (MS) questionnaire by students and teachers.

Regarding the MS as a whole, both samples perceived its implementation as “moderate” (60-89 points; students: M = 77.12, SD = 16.97, faculty: M = 79.43, SD = 17.24) (Figure 1). On average, students thought implementation of the MS was less successful than faculty did, but the effect was quite small (*p* < 0.05, *d* = 0.14). As a result, there are only small differences in the distribution of opinions (Figure 1): slightly more students than faculty viewed the implementation as poor or slight (13.9% vs. 12.4%), while slightly fewer viewed it as good (22.3% vs. 26.7%) or excellent (0.6% vs. 0.8%). These differences were statistically not significant (*p* = 0.64, *w* = 0.04).

### Item analysis

For both students and faculty the dispersion of item means of the MS questionnaire was adequate (Table 2). The range of means was between 1.21 (item 28: “*The course is family-friendly*”) and 2.87 (item 32: “*The course considers the equality of women and men*”), M = 2.08 (SD = 0.38), in the student sample, and between 1.24 (item 6: “*The teachers receive recognition for their work by the faculty*”) and 2.89 (item 27: “*The course encourages students at a professional level*”), M = 2.15 (SD = 0.35), in the faculty sample.

**Table 2:** Means, standard deviations and part-whole corrected discrimination indices of the mission-statement questionnaire items in the student and teacher sample

***Subscale***	***Item***	***Students***		***Teachers***				
		**(*N =*1119)**		**(*N =*258)**				
		***M*(*SD*)**	**Discrimination**	***M*(*SD*)**	***Discrimination***	***t*(1375)**	***p***	***d***
*The Teachers*	1 The teachers are role models for the students.	1.91 (0.91)	0.49	2.34 (0.84)	0.50	−6.84	< 0.0014	0.49
	2 The teachers are in a livelily dialogue with the students and other teachers.	1.80 (0.95)	0.56	2.28 (0.97)	0.62	−7.34	< 0.0014	0.50
	3 The teachers are competent both didactically as well as in terms of content.	2.11 (0.89)	0.55	2.37 (0.87)	0.62	−4.32	< 0.0014	0.30
	4 The teachers provide stimulating feedback.	1.68 (0.90)	0.51	2.24 (0.84)	0.61	−9.08	< 0.0014	0.64
	5 The teachers are persons in charge and in a position of trust for the students.	1.34 (0.98)	0.56	2.18 (0.86)	0.58	−12.65	< 0.0014	0.91
	6 The teachers receive recognition for their work by the faculty.	1.91 (0.82)	0.36	1.24 (0.98)	0.41	11.29	< 0.0014	0.74
	7 The teachers receive recognition for their work by the students.	2.27 (0.82)	0.38	2.37 (0.95)	0.31	−1.74	0.082	0.11
	8 The teachers are willing to develop perpetually.	2.09 (0.84)	0.54	2.55 (0.84)	0.57	−7.94	< 0.0014	0.54
*The Students*	9 The students learn about the physical, mental and social dimensions of health and disease.	2.33 (0.95)	0.50	2.51 (0.84)	0.59	−2.82	0.005	0.20
	10 The students communicate appropriately, sensitively and respectfully with patients.	2.42 (0.71)	0.24	2.55 (0.68)	0.39	−2.69	0.007	0.19
	11 The students learn to consider the individuality of patients in professional decisions.	2.20 (0.80)	0.48	2.25 (0.85)	0.59	−0.93	0.352	0.06
	12 The students act in consideration of ethical principles.	2.52 (0.76)	0.40	2.60 (0.68)	0.50	−1.44	0.149	0.11
	13 The students learn to impart their knowledge to others.	2.16 (1.00)	0.49	2.03 (0.89)	0.56	1.90	0.058	0.14
	14 The students are well prepared for lifelong learning and to develop personally.	2.30 (0.94)	0.58	2.01 (0.92)	0.67	4.58	< 0.0014	0.31
	15 The students learn to think critically in consideration of evidence and to make decisions on that basis.	2.24 (0.95)	0.56	2.25 (0.92)	0.67	−0.18	0.859	0.01
	16 The students know their personal limits.	2.36 (0.88)	0.33	1.91 (0.79)	0.29	7.42	< 0.0014	0.54
	17 The students learn to consider health- economic conditions.	1.96 (0.89)	0.41	1.81 (0.88)	0.35	2.39	0.017	0.17
	18 The students learn skills of self-organization and time management.	2.39 (1.10)	0.39	2.00 (1.00)	0.50	5.14	< 0.0014	0.37
	19 The students master the basics of scientific work.	1.77 (0.95)	0.51	1.43 (0.86)	0.51	5.24	< 0.0014	0.38
	20 The students learn to master the basic medical competencies.	2.35 (0.88)	0.55	2.50 (0.78)	0.61	−2.44	0.015	0.18
	21 The students learn to make differential diagnoses and to develop treatment strategies independently.	2.05 (0.90)	0.52	2.38 (0.76)	0.58	−5.53	< 0.0014	0.40
	22 The students communicate appropriately, sensitively and respectfully with colleagues.	2.56 (0.76)	0.36	2.40 (0.74)	0.36	3.03	0.002	0.21
	23 The students cope straight and adequately with errors.	2.05 (0.85)	0.46	1.94 (0.80)	0.53	1.94	0.052	0.13
*The Curriculum*	24 The course inspires the students for a self-directed learning.	2.35 (0.99)	0.53	2.18 (0.92)	0.63	2.56	0.011	0.18
	25 The course promotes scientific thinking and working.	2.27 (0.99)	0.51	2.09 (1.09)	0.57	2.47	0.013	0.17
	26 The course is accompanied by educational research.	2.04 (0.87)	0.50	1.99 (0.97)	0.40	0.71	0.478	0.05
	27 The course encourages students on a professional level.	2.79 (0.76)	0.47	2.89 (0.58)	0.46	−1.94	0.052	0.15
	28 The course is family-friendly.	1.21 (0.94)	0.41	1.53 (0.79)	0.34	−5.16	< 0.0014	0.37
	29 The course consists of a core curriculum and offers comprehensive electives.	1.24 (1.01)	0.40	1.66 (0.90)	0.47	−6.17	< 0.0014	0.44
	30 The course provides scope for academic qualification.	1.70 (0.94)	0.46	1.86 (1.05)	0.48	−2.41	0.016	0.16
	31 The course is designed and developed jointly by teachers and students.	1.78 (0.97)	0.56	1.97 (1.00)	0.55	−2.73	0.006	0.19
	32 The course considers the equality of women and men.	2.87 (0.87)	0.27	2.70 (0.89)	0.19	2.84	0.005	0.19
	33 The course is patient-oriented.	1.80 (0.81)	0.51	1.88 (0.83)	0.52	−1.46	0.143	0.10
	34 The course offers scope for stays abroad.	1.82 (1.02)	0.32	2.12 (0.86)	0.36	−4.41	< 0.0014	0.32
	35 The course supports the students on a personal level.	2.13 (1.07)	0.54	2.15 (0.81)	0.54	−0.18	0.861	0.02
	36 The course is problem-oriented.	2.08 (0.84)	0.55	2.17 (0.83)	0.58	−1.48	0.138	0.11
	37 The course is interdisciplinary.	2.29 (1.04)	0.36	2.10 (1.00)	0.48	2.59	0.010	0.19

*M* (*SD*) = mean (standard deviation); t-tests were conducted with a Bonferroni corrected α_*crit*_ of 0.05/37 = 0.0014.

Both groups showed a similar dispersion of DREEM mean scores. The overall item mean in the student sample was M = 2.19 (SD = 0.50), slightly higher than the limit of 2, which at the item level in DREEM is deemed to indicate areas requiring improvement [23,24]. Using this criterion in the MS questionnaire as well, 16 of 37 items pertained to areas where students and/or faculty saw deficiencies. Of these 16 items, 8 items (17, 19, 26, 28, 29, 30, 33, 34) pertained to general aspects of the course (for example free time for academic qualification, patient-orientation, family-friendliness), while the other 8 (1, 2, 4, 5, 6, 16, 23, 31) focused more on intra-/interindividual aspects. In particular, items 1, 2, 4 and 5 pertain to relations between students and faculty. While both groups perceived a similar need to improve the external conditions of studying, the relations between students and faculty were seen very differently, as testified by the large effect sizes (see Table 2), which ranged from *d* = 0.49 for item 1 (“*The teachers are role models for the students*”) to 0.91 for item 5 (“*The teachers are persons in charge and in position of trust for the students*”).

In the student sample the discrimination index ranged from 0.24 (item: 10: “*The students communicate appropriately, sensitively and respectfully with patients*”) to 0.58 (item 14: “*The students are well prepared for lifelong learning and to develop personally*”), M = 0.46 (SD = 0.09); only 2 items fell below the reference level of 0.30, while 18 exceeded 0.50. In the faculty sample the discrimination index fell in a similar range, M = 0.50 (SD = 0.11), with the lowest value 0.19 for item 32 (“*The course considers the equality of women and men*”), the highest 0.67 for item 15 (“*The students learn to think critically in consideration of evidence and to make decisions on that basis*”). Only item 32 fell well below the reference level, while 23 of the 37 items had high discrimination values.

### Test analysis

The reliability of the MS questionnaire (37 items) was comparable to that of the longer DREEM (50 items): α = 0.92 for both in the student sample, α = 0.93 vs. 0.94 in the faculty sample. At the subscale level the MS questionnaire showed a similarly high reliability, with values between α = 0.81 and 0.87 (Table 3).

**Table 3:** Scores of DREEM and Mission-statement (MS) questionnaire in the student and teacher sample

***Questionnaire***	***Maximal score***	***Students *(*N = *1119)**			***Teachers *(*N = *258)**		
		***Internal consistency *(**α**)**	***M *(*SD*)**	***Min-Max***	***Internal consistency *(**α**)**	***M *(*SD*)**	***Min-Max***
MS Questionnaire	148	0.92	77.12^***^	10-144	0.93	79.43^***^	20-133
- Total Score (37 Items)			(16.97)			(17.24)	
Teachers	32	0.81	15.11^***^	0-32	0.82	17.57^***^	4-31
(8 Items)			(4.67)			(4.73)	
Students	60	0.83	33.65^***^	6-60	0.87	32.57^***^	10-55
(15 Items)			(7.35)			(7.38)	
Curriculum	56	0.81	28.36^***^	3-55	0.83	29.29^***^	4-49
(14 Items)			(7.06)			(7.02)	
DREEM - Total Score	200	0.92	109.75^***^	28-182	0.94	117.63^***^	52-182
(50 Items)			(21.71)			(20.80)	

α = Cronbach’s α; *M* (*SD*) = mean (standard deviation); *Min-Max* = minimal and maximal score achieved; ^*^p < 0.05; ^**^p < 0.01; ^***^p < 0.001.

The total scores of both DREEM and the MS questionnaire showed a high and significant positive correlation in both the student and faculty sample (*r* = 0.79 and 0.80, respectively, both *p*’s < 0.001).

Educational climate in DREEM and implementation of the MS were perceived more negatively by students than by faculty, but each group gave itself a better grade. On the MS questionnaire, students’ perception of teachers was poorer (M = 15.11, SD = 4.67) than teachers’ perception of themselves (M = 17.57, SD = 4.73; *p* < 0.001, *d* = 0.52), while students’ perception of themselves was more positive (M = 33.65, SD = 7.35) than their teachers’ perception of them (M = 32.57, SD = 7.38; *p* < 0.05, *d* = 0.15). Students and faculty evinced no significant difference in their perception of the course (M = 28.36, SD = 7.06 vs. M = 29.29, SD = 7.02; *p* = 0.06, *d* = 0.13).

Independent-samples t-tests showed that students in the clinical course viewed the implementation of the MS in a significantly more negative light than their colleagues in the preclinical course. There were significant negative correlations between the MS questionnaire and year of study, although none appeared in DREEM (Table 4).

**Table 4:** Correlations of the Mission-statement (MS) questionnaire with DREEM total score and subscales in the student and teacher sample

	***Students***	***Teachers***
	**(*N *= 1119)**	**(*N *= 258)**
	***Correlation with MS questionnaire total score (r)***	
DREEM - Total score	0.79^***^	0.80^***^
Perception of teaching	0.76^***^	0.74^***^
Perception of teachers	0.64^***^	0.64^***^
Academic self-perception	0.68^***^	0.72^***^
Perception of atmosphere	0.66^***^	0.71^***^
Social self-perception	0.41^***^	0.56^***^

^***^p < 0.001.

To assess whether the perceptions of teachers depend on the phase in which they teach, analyses of variance were performed with phase of study as the independent variable (grouped as “preclinical only”, n = 30; “clinical only”, n = 152; “both”, n = 42) and teachers’ responses on both questionnaires as the dependent variable.

Descriptively, it is apparent on most dimensions that teachers in the preclinical course took a more negative view than their clinical colleagues, and an even more negative view than those who teach in both phases (Table 5).

**Table 5:** Teachers’ perceptions of the learning environment in relation to their year of teaching

***Questionnaire***	***Teachers of***			***p***	**η**^**2**^	***Posthoc differences***
	***Preclinical course***	***Clinical course***	***Both courses***			
	**(*N *= 30)**	**(*N *= 152)**	**(*N *= 42)**			
	***M *(*SD*)**	***M *(*SD*)**	***M *(*SD*)**			
MS questionnaire - Total score	76.03 (15.06)	79.25 (15.55)	82.14 (24.01)	0.34	0.01	
Teachers	17.80 (4.44)	17.55 (4.40)	17.50 (6.15)	0.96	0.00	
Students	29.87 (6.76)	32.47 (6.93)	34.57 (9.47)	< 0.05	0.03	preclinical course < both courses
Curriculum	28.37 (5.80)	29.22 (6.22)	30.07 (9.87)	0.59	0.01	
DREEM - Total score	111.53 (17.94)	117.87 (19.32)	120.26 (26.55)	0.20	0.02	

*M* (*SD*) = mean (standard deviation); MS = mission statement; *posthoc-differences* = Tukey’s test at α_*crit*_ = 0.05.

When students had already completed training in another area (such as nursing or geriatric care), this affected their scores on the MS questionnaire. Students with previous training (n = 241) took a significantly dimmer view of the implementation of the MS (M = 72.17, SD = 17.55) than their peers without such training (n = 796) (M = 78.42, SD = 16.46), but effect sizes were small (*p* < 0.001, *d* = 0.37).

Students who were not native speakers (n = 134) thought the MS had been better implemented (M = 85.12, SD = 18.73) than did the native speakers (M = 76.03, SD = 16.43); (*p* < 0.001, *d* = 0.52). The number of non-native speakers in the faculty sample (n = 9) was too small for meaningful inference testing.

Comparing male and female students, it is apparent that women (n = 739, M = 78.06, SD = 16.06) perceived the implementation of the MS significantly more positively than did men (n = 380, M = 75.28, SD = 18.51), but the effect was quite small (*p* < 0.01, *d* = 0.16). No such differences were found in the faculty sample.

To examine the factorial validity of the MS questionnaire, we conducted in both groups (students and teachers) explorative principal components analyses. After successful factorization of the 37 items in the student sample (KMO = 0.94, Bartlett: *p* < 0.001), there were seven factors with eigenvalues > 1. After analysis of the screen plot, two factors were extracted and subjected to an orthogonal rotation. In total, these two factors explained 32.2% of the variance and exhibited a satisfactory simple structure. We interpreted them as “The teachers and the curriculum” and “The students and the curriculum”, i.e. items dealing with the curriculum were not represented by a dimension of its own, but were distributed rather equally among the two remaining subscales (Table 6).

**Table 6:** Factor analytic loadings of the MS questionnaire items in the student sample

***Item***	***I***	***II***
	***“The teachers and the curriculum*”**	***“The students and the curriculum*”**
5 The teachers are persons in charge and in a position of trust for the students.	0.73	
31 The course is designed and developed jointly by teachers and students.	0.64	
2 The teachers are in a livelily dialogue with the students and other teachers.	0.63	
4 The teachers provide stimulating feedback.	0.63	
29 The course consists of a core curriculum and offers comprehensive electives.	0.61	
33 The course is patient-oriented.	0.57	
28 The course is family-friendly.	0.57	
3 The teachers are competent both didactically as well as in terms of content.	0.57	
30 The course provides scope for academic qualification.	0.56	
8 The teachers are willing to develop perpetually.	0.53	
1 The teachers are role models for the students.	0.50	
26 The course is accompanied by educational research.	0.48	
36 The course is problem-oriented.	0.46	0.38
21 The students learn to make differential diagnoses and to develop treatment strategies independently.	0.46	0.34
19 The students master the basics of scientific work.	0.44	0.33
34 The course offers scope for stays abroad.	0.44	
17 The students learn to consider health-economic conditions.	0.41	
6 The teachers receive recognition for their work by the faculty.	0.38	
37 The course is interdisciplinary.	0.31	
27 The course encourages students on a professional level.		0.60
22 The students communicate appropriately, sensitively and respectfully with colleagues.		0.60
12 The students act in consideration of ethical principles.		0.59
14 The students are well prepared for lifelong learning and to develop personally.	0.31	0.59
15 The students learn to think critically in consideration of evidence and to make decisions on that basis.	0.32	0.56
24 The course inspires the students for a self-directed learning.	0.32	0.51
13 The students learn to impart their knowledge to others.		0.51
9 The students learn about the physical, mental and social dimensions of health and disease.		0.51
16 The students know their personal limits.		0.49
11 The students learn to consider the individuality of patients in professional decisions.		0.49
20 The students learn to master the basic medical competencies.	0.38	0.48
23 The students cope straight and adequately with errors.		0.48
10 The students communicate appropriately, sensitively and respectfully with patients.		0.46
32 The course considers the equality of women and men.		0.45
35 The course supports the students on a personal level.	0.40	0.43
25 The course promotes scientific thinking and working.	0.38	0.41
18 The students learn skills of self-organization and time management.		0.40
7 The teachers receive recognition for their work by the students.		0.30

Loadings < 0.30 are not shown for reasons of clarity.

In the teacher sample (KMO = 0.91, Bartlett: *p* < 0.001), factor analysis revealed eight dimensions with eigenvalues > 1. Following the screen plot, a 3-factor-solution was chosen and rotated orthogonally (variance explanation 41.3%). The dimensions identified were interpreted as “Medical goals of the curriculum and the teachers”, “General conditions of the curriculum” and “Students’ social skills” (Table 7).

**Table 7:** Factor analytic loadings of the MS questionnaire items in the teacher sample

***Item***	***I***	***II***	***III***
	***“Medical goals of the curriculum and the teachers*”**	**“*General conditions of the curriculum*”**	**“*Students’ social skills*”**
3 The teachers are competent both didactically as well as in terms of content.	0.76		
8 The teachers are willing to develop perpetually.	0.73		
2 The teachers are in a livelily dialogue with the students and other teachers.	0.66	0.35	
20 The students learn to master the basic medical competencies.	0.64		
9 The students learn about the physical, mental and social dimensions of health and disease.	0.64		
11 The students learn to consider the individuality of patients in professional decisions.	0.61		0.31
4 The teachers provide stimulating feedback.	0.60	0.32	
1 The teachers are role models for the students.	0.59		
15 The students learn to think critically in consideration of evidence and to make decisions on that basis.	0.58		0.41
21 The students learn to make differential diagnoses and to develop treatment strategies independently.	0.57		0.35
5 The teachers are persons in charge and in a position of trust for the students.	0.56	0.32	
24 The course inspires the students for a self-directed learning.	0.54		0.44
36 The course is problem-oriented.	0.52	0.42	
33 The course is patient-oriented.	0.50	0.44	
14 The students are well prepared for lifelong learning and to develop personally.	0.49	0.32	0.40
25 The course promotes scientific thinking and working.	0.45		0.31
13 The students learn to impart their knowledge to others.	0.44		0.40
27 The course encourages students at a professional level.	0.42		
35 The course supports the students on a personal level.	0.42	0.35	
34 The course offers scope for stays abroad.		0.59	
30 The course provides scope for academic qualification.		0.58	0.42
29 The course consists of a core curriculum and offers comprehensive electives.		0.58	
28 The course is family-friendly.		0.57	
17 The students learn to consider health-economic conditions.		0.56	
31 The course is designed and developed jointly by teachers and students.	0.31	0.55	
19 The students master the basics of scientific work.		0.49	0.38
18 The students learn skills of self-organization and time management.		0.46	0.36
26 The course is accompanied by educational research.		0.44	
37 The course is interdisciplinary.	0.35	0.41	
6 The teachers receive recognition for their work by the faculty.		0.34	
22 The students communicate appropriately, sensitively and respectfully with colleagues.			0.67
23 The students cope straight and adequately with errors.		0.32	0.59
10 The students communicate appropriately, sensitively and respectfully with patients.	0.41		0.55
12 The students act in consideration of ethical principles.	0.45		0.48
7 The teachers receive recognition for their work by the students.			0.47
16 The students know their personal limits.			0.46
32 The course considers the equality of women and men.			0.32

Loadings < 0.30 are not shown for reasons of clarity.

## Discussion

The present study demonstrated that a faculty-specific MS “teaching” questionnaire can be useful to measure local features of the educational climate at a unique institution and to highlight discrepancies between nominal and actual conditions as they are perceived by the faculty. The high correlation of our MS “teaching” questionnaire with DREEM indicates the impact of mission statements on measuring the educational climate as proposed by Genn [9]. The development of our MS-“teaching” according to international standards and recommendations for good medical education and practice could certainly be one reason for this finding. One can consequently deduce that good teaching correlates well with a good educational climate.

### Insights beyond DREEM

Our questionnaire provides a reliable instrument to measure the learning climate with a strong focus on competencies which are increasingly considered crucial in medical education and thus offers additional information beyond the DREEM. Our MS “teaching” and the derived questionnaire define explicit targets for competencies (C) such as the Diagnostic and Therapeutic-C, Communicative-C, Social and Ethical-C, Scientific-C, Teaching-C, Economic-C, and Self-C (i.e “*the students know their personal limits*”, “*learn to impart their knowledge to others*”, “*learn to think critically*”). In addition, another focus refers to the interaction between teachers and students (i.e. “*The teachers are persons in charge and in position of trust for the students*”). The very specific feedback on to what extent the defined competencies and interactions are already implemented - in the perception of the faculty - provides an important basis for further faculty and competency development with the students in a trustworthy environment. The acceptance of feedback, for instance, depends on its perceived accuracy and results from a feedback-friendly environment as well the trustworthiness of the person providing feedback [28]. In another study, we demonstrated that the relationship between the person giving feedback and the person receiving feedback is essential to develop a feedback culture [29].

### Psychometric properties

Item and test indices of the MS questionnaire were very good. While item means ranged at a comparatively low level, their dispersions were similarly high in both the student and faculty sample, albeit less than for the DREEM items. Only three items of the MS questionnaire had unsatisfactory discrimination indices [25]. Both questionnaires were overall internally consistent. The high positive correlation between the two questionnaires demonstrates the utility of the MS questionnaire for studying educational climate. Neither in the student nor in the teacher sample did factor analysis perfectly reproduce the three areas of the MS, i.e. “the teachers”, “the students” and “the curriculum”: The subscales could be identified as such, especially among teachers, however, items referring to the curriculum did not load on one exclusive factor, but were rather distributed over two dimensions. Besides, the subscale representing “the students” was mainly characterized by items dealing with students’ social skills. In the student sample, the three areas of the MS were reduced to two dimensions (students vs. teachers) with curriculum items loading highly either on the student or on the teacher dimension. These results emphasize the perceptional differences between students and teachers regarding the MS. Nevertheless, they do not contradict the notion that the MS questionnaire can be analyzed using the three, admittedly rather descriptive than factorial, subscales we refer to in the present paper. Similarly, the DREEM often failed to demonstrate its five-factorial structure – nevertheless, the five original subscales have not been discarded, as they proved useful in examining educational environments [18]. In agreement with other studies [17,18], our students saw themselves in a more positive light than did their teachers, while teachers’ self-image was more positive than their image among students. *In-group bias* is a possible cause of this effect [30]. As is well known from social-psychological research on the *fundamental attribution error*, the effort to maintain their own positive self-image leads most people to criticize others (external attribution) rather than themselves (internal attribution) [31,32], often overrating their own (desirable) characteristics in comparison with the norm (*self-serving bias*) [33,34]. Students had a more negative perception of educational climate and implementation of the MS than did teachers. This is perhaps due to the fact that teachers feel a much greater responsibility for good instruction, and hence for successful implementation of the MS “teaching”, than do students.

From the perspective of students in the clinical phase of their studies the implementation of the MS was seen in a poorer light than by preclinical students. A possible explanation may be due to the fact that the MS places emphasis on clinical and patient-oriented instruction, points that currently are not a prominent part of the preclinical phase and certainly deserves improvement. Preclinical students apparently anticipate a greater clinical relevance in their clinical studies than those who are actually in the midst of their studies. The phase of studies in which faculty members teach had a considerably smaller effect on the results of the faculty sample. This may be explained by the fact that as a rule, a teacher is involved in only one phase of the curriculum and primarily sees his or her own subject, while students move on and can readily make comparisons. Nevertheless, teachers in the preclinical course perceived the implementation of the MS as poorer than their clinical colleagues. This may be due to the MS’s emphasis on patient-orientation, as well as the general lack of teaching in a clinical context as already mentioned.

Another important observation is that not only students who had already completed training in another area, but also teachers who had not studied medicine themselves, had a more negative perception of climate than their counterparts without such experience. The possibility of drawing comparisons (negative or positive) with outside experience may possibly affect the impression of educational climate in medical studies, leading to higher (or lower) expectations. These findings might be taken into account in choosing applicants for medical studies or in curriculum development, for example by designing areas of the curriculum suited to the needs of particular groups.

In agreement with other studies [10,12,18,23,35,36], our data confirm that women have a more positive perception of educational climate than their male colleagues, although we found only small effects. Roff [16] found that sex-specific differences in the perception of educational environments depended also on a number of cultural factors. However, socio-cultural factors seem to play a role independent of a respondent’s sex, since students with other mother tongue perceived a better implementation of the MS, both in general and on the subscales, and also a better educational climate in DREEM. In light of increasing internationalization of courses, such findings might also be useful in developing a curriculum suited to the individual needs of particular groups.

The results of the present study indicate as well that the educational climate in the faculty correlates positively with the degree to which faculty members take note of the MS and put it into practice. Ascertainment of the educational climate is regarded as a necessary first step towards implementation of a reformed curriculum [10,11].

### Suggestions for future improvements

The development of individual, faculty specific questionnaires based on the MS of the faculty can serve to measure the perceived degree to which specific goals have been implemented. Such a survey has been undertaken for the first time in our faculty and the resulting data serve as the basis for measuring the educational climate at various stages of curriculum reform [37].

Teachers feel their work as being held in low esteem within faculty, while students and teachers have very discrepant perceptions. These findings offer opportunities for the systematic optimization of the educational culture. Efforts to enhance the impact of good teaching in promoting academic careers, the introduction of mentoring programs [38], and the implementation of effective feedback [39] could all contribute to forming a true community of teachers and students.

### Critical assessment

The present study has some limitations. First, the implementation of an MS cannot be fully assessed by using solely the perceptions of current students and teachers. Views of other stakeholders such as past students (i.e. graduates), accreditation bodies, external examiners, and employers should also be considered to evaluate the implementation of an MS in an exhaustive manner. This is especially so, since an MS usually involves long term goals. Second, the value of the present results is limited by the fact that not everyone involved, in particular not everyone teaching in the medical faculty, participated in the survey. Just how representative are the results? It might be that teachers participated who used the opportunity to vent their critical opinion. In particular, the item on the MS questionnaire with the lowest mean (item 6: “*The teachers receive recognition for their work by the faculty*”) indicates that this might be the case. On the other hand, faculty members who are very interested in teaching can be adjudged to be highly motivated to participate in the survey. The student sample, with a larger number of respondents, is less affected by such issues. To ensure validity, as many as possible should participate, but this is difficult to achieve as long as participation is voluntary. Third, the MS questionnaire is not fully able to measure the implementation of goals described in the MS. Similarly to DREEM, a questionnaire can only assess the perceived, i.e. subjective, implementation, especially since some of the goals of the MS “teaching” can hardly be assessed objectively, i.e. “*The teachers are in charge and persons in position of trust for the students*” or “*The atmosphere is relaxed during classes*” (example from DREEM). Both for the curriculum and for faculty development, however, this sort of information is important – maybe even more important than purely objective data.

Fourth, our questionnaire, derived from the MS “teaching” of our faculty, may at first sight seem to be an instrument which only fits our faculty. MSs from other faculties may have different orientations, which makes our questionnaire not necessarily transferable. Note however that these faculties may develop their own questionnaires from their specific MSs in the same methodological manner as we did. Besides, our questionnaire provides a reliable instrument to measure the learning climate focusing - more strongly than DREEM - on competencies, which is why it can be useful to the increasing number of faculties running or planning a competency-based curriculum.

## Conclusions

The perceived implementation of faculty-specific goals can be measured in an institution to some considerable extent by means of a questionnaire developed on the basis of the institution’s MS. The correlations between our MS questionnaire and DREEM suggest that the perceived implementation of a MS for medical education is a good indicator of the educational climate in the faculty, too. Together with DREEM as a general and internationally validated instrument for measuring educational climate, locally and site-specifically developed MS questionnaires provide the foundation for defining future improvements concerning local features within unique faculties. Repeated measurements enable the assessment of progress in realizing the goals defined in the MS. Additionally, our MS questionnaire showing strong psychometric properties might prove useful to the increasing number of faculties seeking to measure their learning climate as well as their perceived competency orientation in a competency-based curriculum.

### Ethical approval

Ethical approval was given by the independent ethics committee of the Medical Faculty, Heinrich-Heine-University Düsseldorf.

## Competing interests

The authors declare that they have no competing interests.

## Authors’ contributions

TR had the idea for the project. He was involved in the development of the study design, questionnaire and analysis of data. He wrote the article. MSO was in involved in study design and the development of the questionnaire. He analyzed the data and wrote the article. JDB performed the online survey and she was responsible for data preparation. She revised the article critically. KDK, UD, MS and SRT were involved in study design and they revised the article critically. All authors read and approved the final manuscript.

## Authors’ information

TR, Dr. med, MME is a senior physician for internal medicine/diabetics and master of medical education. He is head of the clinical skills center and speaker of the curriculum development group.

MSO, Dr. rer. nat. is a psychologist and researcher at the institute of experimental psychology. He is also a medical student.

KDK, Prof. Dr. rer. nat. is professor of biochemistry and master of medical education. He is speaker of the curriculum development group.

JDB, Dr. rer. nat. is a psychologist and responsible for evaluation at the Medical faculty.

UD, Prof. Dr. med. is professor in physiology and deputy dean of study for the preclinical part.

MS, Prof. Dr. med. is a full professor of medicine and head of the rheumatology sector. He is deputy dean of study for the clinical part.

SRT, Prof. Dr. med is a full professor of medicine and head of the institute for forensic medicine. She is the dean of study at Medical faculty.

## Pre-publication history

The pre-publication history for this paper can be accessed here:

http://www.biomedcentral.com/1472-6920/12/109/prepub
